# Twin Pregnancies in Dairy Cattle: Incidence, Reproductive Performance, and Farm-Level Economic Impact in a Red Holstein Herd in Romania

**DOI:** 10.3390/ani15223284

**Published:** 2025-11-13

**Authors:** Daniel Berean, Raluca Cimpean, Liviu Marian Bogdan, Ionela Ut, Stefan Coman, Simona Ciupe, Sidonia Gog Bogdan

**Affiliations:** 1 Department of Reproduction, Faculty of Veterinary Medicine, University of Agricultural Sciences and Veterinary Medicine of Cluj-Napoca, Calea Manastur 3–5, 400372 Cluj-Napoca, Romania; daniel.berean@usamvcluj.ro (D.B.); liviu.bogdan@usamvcluj.ro (L.M.B.); ionela-delia.ut@student.usamvcluj.ro (I.U.); coman.stefan25@yaho.com (S.C.); simona.ciupe@usamvcluj.ro (S.C.); 2 Department of Animal Breeding and Food Safety, Faculty of Veterinary Medicine, University of Agricultural Sciences and Veterinary Medicine of Cluj-Napoca, Calea Manastur 3–5, 400372 Cluj-Napoca, Romania; 3 Department of Surgery, Anesthesiology and Intensive Therapy, University of Agricultural Sciences and Veterinary Medicine of Cluj-Napoca, 400372 Cluj-Napoca, Romania; sidonia.bogdan@usamvcluj.ro

**Keywords:** twinning, dairy cattle, reproductive performance, milk yield, freemartinism, economic losses

## Abstract

This study evaluated the biological and economic impact of twin calvings in a commercial dairy herd located in Harghita County, Romania. Data from 2019 to 2022 were analyzed retrospectively using production, reproductive, and veterinary records. The incidence of twin pregnancies averaged 11.0% across the study period. Mixed-sex pairs were most frequent (55.3%), followed by female–female (28.2%) and male–male (16.5%) combinations. Twin calvings were associated with significant reductions in subsequent milk yield (−742 kg per lactation; *p* < 0.05), decreased fertility (2.3 vs. 1.4 inseminations; 103 vs. 79 days service period; *p* < 0.05), and higher veterinary costs (EUR 90 per case). The expected loss of replacement value due to freemartinism was EUR 63 per twin calving. After accounting for a small gain in calf value, the total economic loss was estimated at EUR 379 per twin calving. These findings demonstrate that twin pregnancies, although relatively uncommon, substantially reduce productive efficiency and profitability in dairy systems. Early detection and targeted reproductive management are recommended to mitigate their adverse effects and enhance farm sustainability.

## Simple Summary

Twinning in dairy cattle may seem advantageous because it produces two calves at once, but in dairy herds, it often causes serious problems. This study examined twin calvings in a dairy herd in Romania over three years. Twin pregnancies occurred in 11% of all calvings and were linked with lower milk yield after calving, poorer fertility, and higher veterinary costs. Cows with twins produced 742 kg less milk per lactation, needed more inseminations, and took longer to become pregnant again. More than half of twin pairs were mixed-sex, meaning many female calves were freemartins and unable to reproduce. Altogether, each twin calving caused an estimated loss of about EUR 379. These results show that twinning has clear negative effects on both animal performance and farm profitability. Managing and detecting twin pregnancies early can help reduce these losses and improve herd efficiency.

## 1. Introduction

Twinning in dairy cattle represents a reproductive anomaly with wide-ranging biological and economic implications [1]. While twin births may be perceived as beneficial in beef cattle systems, where additional calves directly contribute to higher profitability, in dairy herds, such events are considered undesirable due to the cascade of complications they trigger [2,3]. Globally, the incidence of twin calvings in dairy cattle ranges from approximately 2% to 5% of all pregnancies, with higher rates reported in high-producing Holstein cows [1,2]. Approximately 97% of twins are dizygotic, meaning they result from double ovulations [4]. High feed intake associated with high milk production in dairy cows leads to increased hepatic metabolism of steroid hormones and decreased blood concentrations. Low progesterone concentrations at the time of follicle deviation are a major cause of double ovulations, which can result in twin pregnancies [5].

Twin gestations are strongly associated with increased periparturient health risks, reduced longevity of cows, and impaired calf viability. Documented adverse outcomes include higher incidences of dystocia, retained placenta, metritis, metabolic diseases, and diminished fertility rates, all of which negatively influence both productive lifespan and profitability [6,7,8].

From a herd management perspective, the impact of twinning extends beyond immediate health complications. Twin pregnancies reduce reproductive efficiency by prolonging the service period, increasing the number of inseminations required per conception, and lengthening calving intervals [7,9]. Together with decreased milk yield and lower calf survival rates, these inefficiencies generate measurable economic losses. A further complication is the frequent occurrence of freemartinism in female calves from mixed-sex twin pairs, which eliminates their reproductive potential and diminishes their long-term economic value [9,10,11].

International studies have identified genetics, parity, seasonality, nutritional status, and high milk yield as key risk factors for twinning. High-producing dairy cows, particularly in intensive production systems, are predisposed to multiple ovulations due to complex interactions between metabolism and endocrine regulation [12,13]. Although extensively investigated globally, there remains a notable gap in understanding the incidence and economic consequences of twinning. Farm-level analyses are particularly valuable because they provide context-specific insights needed to refine reproductive management practices.

Studies estimate that each twin calving can add around USD 100 in additional costs due to increased veterinary treatments, assisted calvings, and prolonged recovery periods [9]. Furthermore, milk yield reductions of 3–5% per lactation have been reported in cows experiencing twin pregnancies, translating into significant cumulative losses in high-yielding herds [10]. Calves from twin gestations also weigh less at birth and have higher neonatal mortality, lowering their market value. In the case of mixed-sex twin pairs, the prevalence of freemartinism, affecting approximately 82–97% of female calves, renders them infertile, representing a direct loss of future breeding stock [11,14,15,16]. Taken together, these factors illustrate how twinning not only compromises reproductive performance but also directly undermines farm profitability.

The present investigation aimed to determine the incidence of twin pregnancies in a commercial dairy herd, while explicitly integrating biological outcomes with farm economics and reproductive management. By linking reproductive efficiency indicators, milk production parameters, and direct economic costs, this study sought to provide a holistic assessment of how twinning influences both herd performance and the financial sustainability of dairy enterprises.

## 2. Materials and Methods

### 2.1. Study Design and Farm Description

This investigation was designed as a retrospective observational study conducted on a commercial dairy farm located in the Ciuc Depression, Harghita County, Romania (approximate coordinates: 46.3500° N, 25.8000° E). The farm was established in 2007 and maintains a Red Holstein herd with a well-documented calving history, providing a robust dataset for reproductive and economic analysis. The Red Holstein breed is recognized for its high milk production potential and good adaptability to intensive dairy systems, with average lactation yields ranging from 7000 to 9500 kg and milk fat content between 3.6% and 4.2%. During the study period (2019–2022), the herd size increased from approximately 300 to nearly 400 lactating cows. A total of 773 calvings were recorded over the four-year study period, and the cows included in the study were evenly distributed across parities, with a balanced representation of first-lactation and multiparous cows, ensuring uniformity in reproductive performance assessment and minimizing parity-related bias in the evaluation of twin pregnancies and their associated economic outcomes. The Red Holstein population on this farm originated from imported heifers and semen sourced from high-index breeding programs in Germany and the Netherlands, selected for milk yield, udder conformation, and reproductive performance. Since herd establishment, continuous genetic improvement has been pursued through artificial insemination using proven Red Holstein sires with balanced breeding values for production and functional traits. Artificial insemination of the cows is performed 8–12 h after estrus detection by the milking machine. A veterinarian specialized in reproduction visits the farm monthly to confirm pregnancies and detect reproductive pathologies using ultrasonography.

### 2.2. Housing and Management

Cows were housed in free stall barns with individual resting boxes. Lactating cows were managed in a barn of 1870 m^2^ capacity equipped with two Lely Astronaut robotic milking units, which recorded production- and reproduction-related parameters continuously. Prepartum cows, dry cows, and heifers were housed in separate barns under appropriate group housing systems. Calves up to 2 weeks of age were kept in individual pens, after which they were transferred to collective pens by sex and age category. Feeding was based on a total mixed ration (TMR), with concentrates supplemented through the robotic milking system. Approximately 50% of the forage base was supplied from farm-owned resources, with concentrates sourced externally.

### 2.3. Study Period and Data Sources

The analysis covered three consecutive calendar years, from January 2019 to December 2022. Reproductive and productive data were collected from two complementary sources: the robotic milking system database, which automatically records daily milk yield, milking frequency, and individual performance indicators; and the farm’s gynecological registers and veterinary records, which documented calving outcomes, reproductive interventions, and health treatments.

The study focused on both reproductive and productive parameters associated with twin pregnancies. Reproductive indicators included the incidence of twin calvings, gestation length, conception rate, number of inseminations per conception, and length of the service period (the interval between calving and successful conception). Productive parameters comprised daily and total lactation milk yield, with comparisons drawn between singleton and twin parturitions.

### 2.4. Economic Framework

Economic evaluation of twin calvings was conducted by integrating biological outcomes with standardized monetary values, expressed in euros (EUR). The analysis included four main cost categories: (1) direct production losses, (2) reproductive inefficiencies, (3) health and veterinary costs, and (4) replacement value risk associated with freemartinism.

Direct production losses were estimated from the combined effects of reduced milk yield and altered calf market value. The mean reduction in milk yield following twin calving, determined from herd production records, was multiplied by the average farm gate milk price (EUR 0.335 per L; midpoint of EUR 0.32–0.35). Calf value was calculated by comparing the expected revenues from singleton calvings with those from twin calvings. For a singleton, the expected revenue was EUR 225 per calf. In the case of twin calvings, each calf was valued at EUR 170, giving a total of EUR 340 per twin calving. This results in a partial gain of EUR 115 per twin calving compared to a single calf. In the overall economic analysis, this additional revenue from twins was treated as a negative cost when calculating the total economic loss associated with twin pregnancies, as it partially offsets other costs such as increased veterinary interventions, reproductive problems, or management challenges (Table 1).

Reproductive inefficiencies were assessed by including the additional insemination required and the longer service interval following twin calvings. Each insemination was valued at EUR 22.50, and each extra day open (mean 24 days) was assigned a cost of EUR 3 per day, following standard dairy herd economic models [9].

Health and recovery costs were estimated at EUR 90 per calving, representing the combined expenses of assisted delivery and postpartum veterinary treatment. Replacement value risk was incorporated to account for the loss of reproductive potential in female calves affected by freemartinism. The economic differential between a fertile replacement heifer and a freemartin heifer was estimated at EUR 115 per case, reflecting the average loss in replacement value per twin calving due to the presence of mixed-sex pairs (Table 1).

All costs were expressed on a per-twin-calving basis. The total economic loss was calculated as the sum of all cost components minus the calf value gain. This integrative approach provided a comprehensive assessment of the financial implications of twinning under commercial dairy conditions.

### 2.5. Statistical Analysis

Descriptive statistics were calculated for all parameters and are presented as means ± standard deviation (SD) for continuous variables or as percentages for categorical variables. The normality of data distribution was assessed using the Shapiro–Wilk test. Differences between singleton and twin pregnancies were analyzed using Student’s *t*-test for normally distributed variables and the Mann–Whitney U test for non-normally distributed data. Categorical variables such as sex ratio and calving ease were compared using the chi-square (χ^2^) test.

To evaluate factors associated with the occurrence of twin pregnancies, a binary logistic regression model was applied, with calving type (singleton = 0, twin = 1) as the dependent variable. Since multiple calvings could originate from the same cow, potential non-independence of observations was tested. Where repeated calvings were present, results were verified using a generalized estimating equation (GEE) approach to account for within-cow correlation.

Model assumptions were checked by examining residuals, outliers, and multicollinearity statistics (tolerance and variance inflation factor). Statistical significance was established at *p* < 0.05. All analyses were performed using IBM SPSS Statistics for Windows, Version 28.0 (IBM Corp., Armonk, NY, USA).

## 3. Results

### 3.1. Annual Incidence of Twin Pregnancies

During the four-year study period (2019–2022), a total of 773 calves were born, comprising 85 twin calvings and 603 singleton calvings. The annual incidence of twin births remained relatively stable, fluctuating between 9.82% and 11.66%, without a statistically significant trend over time (χ^2^ = 0.51, *p* = 0.917). In 2019, the herd comprised approximately 300 lactating cows, and twin calvings accounted for 10.98% of all births. A slight decline was observed in 2020 (9.82%), which may be associated with minor variations in herd management and nutrition. As herd size expanded in 2021 and 2022, the number of calvings increased considerably, and the proportion of twin births also rose modestly, reaching its highest level of 11.66% in the final year of observation. These findings suggest a consistent reproductive pattern across years, indicating that herd expansion and management adjustments did not substantially alter the overall frequency of twin calvings during the study period (Table 2).

### 3.2. Distribution of Twin Calvings by Sex

The sex distribution of twin pairs was as follows: 14 male–male pairs (16.47%), 24 female–female pairs (28.23%), and 47 mixed-sex pairs (55.29%). The statistical determinations revealed a highly significant deviation (χ^2^ = 20.21, *p* < 0.05), with mixed-sex twins occurring at a much higher frequency than either same sex combination. This result confirms the biological tendency, widely reported in cattle, for heterologous sex combinations to be more prevalent in twin births (Table 3).

### 3.3. Comparison of Average Milk Yield According to Calving Type

The results summarized in Table 4 demonstrate a clear and consistent reduction in milk yield for cows calving twins compared with those calving a single calf over the four-year study period (2019–2022). The average milk yield per cow following twin calvings was 8991.3 kg, while cows with single calvings produced 9733.3 kg on average, resulting in an overall mean difference of −742 kg per cow. This difference is statistically significant (*p* < 0.05), confirming that twin pregnancies have a negative impact on subsequent lactation performance. A closer examination of the yearly data shows that the reduction in milk yield is relatively stable, ranging from −702 to −777 kg per cow per year, with all differences being statistically significant (*p* < 0.05). This consistency suggests that the effect of twin calvings on milk production is robust across different years and is unlikely to be influenced by short-term environmental or management variations within the herd.

### 3.4. Number of Inseminations and Service Period Following Single vs. Twin Calvings

**Figure 1:**
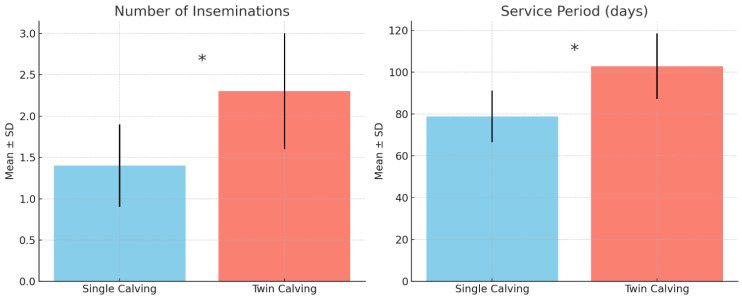
Number of inseminations and service period following single (*n* = 688) vs. twin calvings (*n* = 85). (**Left panel**) number of inseminations, showing a significantly higher mean for twin calvings (*p* < 0.05) (* marked). (**Right panel**) service period (days), also significantly longer (*p* < 0.05) for twin calvings (* marked).

Reproductive performance of cows following single and twin calvings was evaluated by analyzing the number of inseminations required to achieve a subsequent pregnancy and the duration of the service period. Cows that calved a single calf required fewer inseminations on average to conceive again compared with cows that calved twins. The mean number of inseminations after a single calving was 1.4 ± 0.5, whereas cows calving twins required 2.3 ± 0.7 inseminations (Figure 1). The difference between the two groups was statistically significant (*p* < 0.05), indicating that twin calvings are associated with decreased reproductive efficiency and a greater requirement for inseminations to achieve a subsequent pregnancy. The service period, defined as the interval between calving and successful conception, was also affected by calving type. Cows calving a single calf had a mean service period of 78.9 ± 12.3 days, while cows calving twins exhibited a longer mean service period of 102.9 ± 15.7 days. This difference was statistically significant (*p* < 0.05), reflecting the increased reproductive challenges associated with twin calvings.

### 3.5. Detailed Economic Breakdown of Twin Calvings

Reduced milk yield represented the largest source of economic loss. Based on the mean difference of −742 kg of milk per cow between twin and single calvings (Table 3) and the average farm gate milk price of EUR 0.335 per L, the resulting loss was estimated at EUR 262.97 per twin calving.

Reproductive inefficiency was quantified from the observed differences in post calving fertility. Cows following twin calvings required 0.9 additional inseminations on average (2.3 vs. 1.4 inseminations; *p* < 0.05) and had a 24-day longer service period (102.9 vs. 78.9 days; *p* < 0.05). Using a standardized value of EUR 22.50 per insemination and EUR 3 per day open, this equated to a combined loss of EUR 92.25 per twin calving (EUR 20.25 + EUR 72.00).

Health and recovery costs were estimated at EUR 90 per case, representing the average expenditure for assisted delivery, veterinary treatment of dystocia, and management of postpartum disorders.

Replacement value risk due to freemartinism was incorporated by assuming that 55.3% of twin pairs were mixed-sex (Table 2). Given an average loss of EUR 115 per affected female calf, the expected replacement value loss per twin calving was EUR 115 × 0.55 = EUR 63.25.

Calf market value was calculated as the difference between the expected income from a singleton calving (EUR 225) and that from a twin calving (EUR 340 for two calves at EUR 170 each), providing a partial gain of EUR 115 per twin calving. This gain was treated as a negative cost in the overall calculation.

Summing up all components yielded an estimated net economic loss of EUR 379.07 per twin calving, as summarized in Table 5. This value represents the average direct and indirect economic burden associated with twinning in Red Holstein dairy cows under commercial management conditions.

## 4. Discussion

The present study provides an integrated evaluation of the biological and economic implications of twin calvings in a commercial dairy herd. By combining herd-level production and reproductive data with standardized economic assessments, the analysis confirms that twin calvings, although relatively infrequent, exert a disproportionately negative impact on both herd performance and farm profitability. The overall incidence of twinning observed in this study (11.0% across four years) aligns with the upper range of values reported for high-producing dairy cattle. International studies have documented twinning rates of approximately 3–5% in Holstein–Friesian populations [17,18], with higher incidences typically associated with multiparous and high-yielding cows [17]. The comparatively elevated frequency recorded in the present herd may reflect the influence of intensive nutritional regimens, optimized reproductive management, and genetic selection favoring superior milk production, which are factors collectively known to enhance the likelihood of multiple ovulations and twin gestations in dairy cattle.

The sex distribution of twins showed a significant deviation from the expected 1:1:1 ratio, with mixed-sex pairs (55.3%) occurring at a markedly higher frequency than either male–male (16.5%) or female–female pairs (28.2%). This finding aligns with earlier reports by Echternkamp and Gregory (2002) [19], who demonstrated a consistent biological bias toward heterologous sex combinations in cattle twins. The high proportion of mixed-sex pairs carries important management implications, since freemartinism renders the majority of female calves from these pairs infertile, thus eliminating their value as replacements [11]. In this study, the expected economic penalty from freemartinism alone accounted for over EUR 60 per twin calving, underscoring the practical importance of sex distribution in the economic evaluation of twinning.

Twin calvings were associated with an average reduction of 742 kg of milk per lactation compared with singletons. This loss, representing approximately 8% of total yield, is consistent with values reported in other Holstein herds, where López-Gatius et al. (2020) [20] observed decreases ranging between 500 and 1000 kg depending on parity and lactation stage. The biological basis for this reduction is multifactorial, including shorter gestation length, increased metabolic stress during late pregnancy, and higher risk of postpartum disorders such as retained placenta and metritis [21,22,23]. Notably, the effect was consistent across years in this herd, suggesting that environmental variation and management adjustments had limited capacity to offset the negative impact of twin gestations. From an economic perspective, the milk yield deficit alone contributed around EUR 250 per calving to the overall financial loss. While this figure might appear modest in isolation, it becomes critical when considered alongside reduced calf value, reproductive inefficiency, and veterinary expenses. Moreover, reduced persistency in lactation following twin calvings has been documented elsewhere [24], suggesting that the long-term productive lifespan of affected cows may also be compromised.

Reproductive performance following twin calvings was significantly impaired, with affected cows requiring more inseminations (2.3 vs. 1.4) and experiencing longer service periods (103 vs. 79 days). These findings are consistent with the well-established negative association between twinning and fertility. Wiltbank et al. (2002) [25] demonstrated that uterine involution is delayed in cows carrying twins, while increased incidence of postpartum disorders prolongs the interval to first ovulation. In addition, twin calvings are often followed by reduced luteal function and lower conception rates, particularly when metabolic stress is severe [26,27]. The economic consequences of these reproductive inefficiencies are substantial. In this herd, prolonged service periods translated into a calculated opportunity loss of more than EUR 90 per calving, representing nearly one quarter of the total economic burden. This highlights that reproductive costs are not merely indirect consequences but major contributors to the profitability gap between single and twin calvings.

The integrative economic model applied in this study estimated a total loss of EUR 379 per twin calving, which is comparable to values reported in Western European and North American herds [28,29]. Importantly, the breakdown of costs revealed that direct production deficits (milk yield and calf value) accounted for more than half of the total (66%), while reproductive inefficiencies, health interventions, and freemartinism jointly contributed to the remaining 44%. This partition underscores that the economic burden of twin calvings extends beyond immediate productivity losses and is amplified by long-term reproductive and genetic consequences.

The findings of the present study should be interpreted in light of certain limitations. First, the analysis was based on a single commercial herd, which may restrict the generalizability of results to other breeds and management systems. Twinning dynamics in high-yielding Holstein populations, for instance, are well-documented to have increased in parallel with rising milk production [15,17]. In such herds, the biological and economic consequences of twinning may differ in magnitude, particularly given the higher metabolic load and reproductive interventions typical of intensive Holstein management. The literature indicates that, although twin pregnancies may offer potential benefits such as shorter gestation and additional calf income, they consistently compromise milk yield, increase dystocia, perinatal mortality, and metabolic disorders, while also raising culling risks and shortening productive lifespan [21,26,30]. Economic estimates from large-scale studies in Holstein cows have reported losses ranging between USD 59 and 161 per twin calving [29]. These values are considerably lower than those observed in the present study, where the total economic loss per twin calving was calculated at approximately EUR 379. Although higher than previous international estimates, these results remain realistic because they incorporate farm-specific parameters such as local milk price, reproductive performance, veterinary costs, and the particularly high replacement value of heifers. Thus, while the exact magnitude of the loss will vary depending on management system, breed, and market conditions, our findings support the broader conclusion that twin pregnancies consistently impose a substantial economic burden on dairy enterprises. Another limitation is that the present work did not differentiate between unilateral and bilateral twin pregnancies, despite evidence that economic losses are greater for unilateral twins due to increased risk of embryonic loss and perinatal complications [19]. Likewise, we did not investigate the hormonal or metabolic drivers of double ovulation, which are central to the pathogenesis of dizygotic twinning. Future research in Romanian herds should integrate endocrine- and metabolic-profiling with reproductive records to elucidate risk factors and identify cows predisposed to twinning.

From a management perspective, our study did not evaluate intervention strategies following early diagnosis of twin pregnancies. International evidence suggests that management options include leaving the gestation to progress naturally, terminating the pregnancy, attempting manual embryo reduction, or increasing progesterone concentration by supplementation with exogenous progesterone [31]. Recent economic analyses indicate that manual embryo reduction can decrease economic losses compared with no intervention [29]. Incorporating such strategies into herd reproductive management protocols in Romania may offer a promising approach to mitigating losses associated with twinning. Future work should therefore assess the feasibility, economic benefit, and animal welfare implications of adopting these practices in local production systems. Targeted management interventions, such as early identification of freemartin heifers, timely culling, and enhanced reproductive monitoring, are therefore recommended to mitigate these cumulative losses and support long-term economic resilience. The findings of this study reinforce the necessity of integrating reproductive monitoring with economic evaluation when addressing twinning in dairy herds. Early detection through ultrasonography [13] allows for nutritional adjustments and proactive management during late gestation, which may mitigate some of the health and reproductive complications. Additionally, genetic selection against high twinning predisposition has also been proposed [29].

## 5. Conclusions

Twin calvings in the investigated Red Holstein dairy herd occurred at a relatively high incidence (≈11%) and were consistently associated with adverse productive and reproductive outcomes. Cows that delivered twins produced, on average, 742 kg less milk per lactation compared with those calving singletons, required more inseminations to conceive again (2.3 vs. 1.4), and experienced prolonged service periods (103 vs. 79 days). In addition, twin calvings were linked with increased veterinary costs (approximately EUR 90 per case) and a high prevalence of freemartinism (55% of twin pairs), which further reduced the reproductive value of female offspring.

The overall estimated economic loss attributable to twinning was EUR 379 per twin calving, underscoring its significant negative impact on herd profitability. Despite the apparent advantage of producing two calves, the cumulative biological and economic consequences of twinning clearly outweigh potential benefits under commercial dairy production conditions.

Effective mitigation should therefore focus on early identification of twin pregnancies, implementation of adapted reproductive management strategies, and consideration of preventive measures, such as genetic selection and nutritional control, to minimize the occurrence and impact of twin gestations. Continuous monitoring and farm-specific economic evaluations are essential to support decision making and enhance the long-term sustainability of dairy enterprises.

## Figures and Tables

**Table 1:** Input parameters used for estimating the economic loss per twin calving.

Parameter	Base Value	Unit	Source/Reference
Average milk price	0.335	EUR/L	Farm accounting data; regional market average
Calf value (singleton)	225	EUR per calf	Farm sale records
Calf value (twin, per calf)	170	EUR per calf	Farm sale records
Additional insemination cost	22.50	EUR per service	Farm reproductive management data
Cost per extra day open	3.00	EUR per day	Standard dairy herd economic model [9]
Health and veterinary cost	90	EUR per calving	Veterinary service invoices
Replacement value loss due to freemartinism	115	EUR per case	Herd replacement value estimation

**Table 2:** Annual incidence of twin calvings in a Red Holstein herd.

Year	Herd Size	Total Calvings	Twin Calvings	Twin Incidence (%)
2019	300	164	18	10.98
2020	320	163	16	9.82
2021	350	206	23	11.17
2022	400	240	28	11.66
Overall	—	773	85	10.99

Incidence was calculated as the proportion of twin calvings relative to the total number of calvings each year. Confidence intervals (95% CI) for each year were calculated to reflect the variability in twin calving rates. Statistical evaluation using the chi-square test for trend showed no significant difference across years (χ^2^ = 0.51, *p* = 0.917).

**Table 3:** Distribution of twin calvings by sex.

Sex Combination	Observed (*n*)	Expected (*n*)	Chi-Square Contribution
Male–male ^a^	14	28.33	7.25
Female–female ^a^	24	28.33	0.66
Mixed-sex ^b^	47	28.33	12.30
Total	85	85.00	20.21

Values with different superscripts (^a^,^b^) differ significantly (*p* < 0.05). Expected frequencies were calculated under the null hypothesis of equal distribution across sex combinations.

**Table 4:** Comparison of average milk yield according to calving type.

Year	Single Calving (kg, Mean ± SD)	Twin Calving (kg, Mean ± SD)	Difference (kg)	*p*-Value
2019	9728 ± 168	8951 ± 212	−777	0.032
2020	9713 ± 175	9011 ± 205	−702	0.045
2021	9754 ± 162	9022 ± 198	−732	0.038
2022	9738 ± 170	8981 ± 189	−757	0.034
Mean (2019–2022)	9733.3 ± 17.2	8991.3 ± 31.9	−742	<0.05

Mean milk yield (kg) per lactation for cows with single and twin calvings during the 2019–2022 period. Differences represent the reduction in milk yield associated with twin calvings relative to single calvings. Statistical significance was assessed using the independent samples *t*-test; values with *p* < 0.05 were considered significant.

**Table 5:** Detailed economic breakdown of twin calvings.

Cost Category	Component Description	Calculation Basis	Value (EUR)	Direction
Milk yield reduction	742 kg × EUR 0.335 per L	EUR 248.57	−248.57	Loss
Reproductive inefficiency	0.9 inseminations × EUR 22.50 + 24 days × EUR 3 per day	EUR 92.25	−92.25	Loss
Health and recovery	Assisted calving + treatment	EUR 90.00	−90.00	Loss
Replacement value risk	55.3% × EUR 115 replacement loss	EUR 63.25	−63.25	Loss
Calf market value	(2 × EUR 170) − EUR 225 = EUR 115	−115.00 (gain)	+115.00	Gain
Total economic impact	-	-	−379.07	Loss

The model highlights that reduced milk yield accounted for approximately 66% of total losses, followed by reproductive inefficiency (24%), health costs (8%), and replacement value risk (2%). Despite the partial economic gain from producing two calves, the overall effect of twin calving remained strongly negative. These findings emphasize that twin pregnancies, although biologically intriguing, represent a significant economic disadvantage under typical dairy production systems.

## Data Availability

Data are contained within the article.
